# Prematurity and Related Biochemical Outcomes: Study of Bone Mineralization and Renal Function Parameters in Preterm Infants

**DOI:** 10.1155/2011/740370

**Published:** 2011-10-18

**Authors:** Sarika Singh Chauhan, Purnima Dey Sarkar, Bhawna Bhimte

**Affiliations:** Department of Biochemistry, Mahatma Gandhi Medical College, Indore, India

## Abstract

Preterm is defined as a baby with a gestation of less than 37 completed weeks. In this study, serum calcium, phosphorus, ALP, creatinine, and electrolytes were measured in preterm babies. The present study comprised of 75 preterm babies of which 25 were of 28–30 weeks, 25 were of 30–32 weeks, and remaining 25 were of 34–36 weeks (controls) of gestational age. Serum calcium and phosphorus levels were found to be significantly decreased, and serum ALP, creatinine, and electrolytes were found to be significantly increased (*P* < 0.001) at 28–30 weeks as compared to controls, but serum calcium and phosphorous levels were found to be insignificantly decreased, whereas serum ALP activities were found to be insignificantly increased at 28–30 weeks as compared to 30–32 weeks of gestational age in preterm babies. It can be concluded that high serum ALP activity and low serum calcium and phosphorus levels are associated with preterm babies. A significant difference in the mean values of these renal function parameters was also obtained, except for serum sodium and potassium.

## 1. Introduction

Preterm is defined as a baby with a gestation of less than 37 completed weeks (up to 36 weeks or less than 259 days) [1]. A “premature” infant is one that has not yet reached the level of fetal development that generally allows life outside the womb. In the normal human fetus, several organ systems mature between 34 and 37 weeks, and the fetus reaches adequate maturity by the end of this period [2].

Preterm birth is a high risk factor for perinatal morbidity, mortality, and later on neurodevelopmental disabilities and adverse respiratory outcome [3]. Although, the rate of premature birth appears to vary by geographic region, the reported incidence varies between 6 and 10% [4]. Maternal medical conditions increase the risk of PB, and often labor has to be induced for medical reasons; such conditions include high blood pressure [5], preeclampsia [6], maternal diabetes [7], asthma, thyroid disease, and heart disease [8]. An increased risk of prematurity has been noticed among mothers who had a history of previous abortion and a history of previous twin pregnancy [9]. Worldwide, prematurity accounts for 10% of neonatal mortality or around 500,000 deaths per year [10]. The incidence of preterm deliveries in India is 14.5% [11]. Premature infants are known to be at risk of developing metabolic bone disease [12]. Metabolic bone disease is characterized by a failure of complete mineralization of osteoid and encompasses disturbances ranging from mild under mineralization (osteopenia) to severe bone disease with fractures (rickets). MBD is common (50–60%) in infants with 28 weeks of gestation and in those with birth weights 1000 g or less. In these infants, the cause is usually inadequate Ca and phosphate intake. The risk of MBD is inversely proportional to gestational age and birth weight and directly related to postnatal complications [13]. 

As to the biochemical analysis, serum calcium can have normal or low levels, serum phosphorus is low, and the activity of alkaline phosphatase increases as well as that of osteocalcin. Biochemical indicators, such as alkaline phosphatase, have been considered to identify preterm infants with metabolic bone disease. The increase in alkaline phosphatase, with a cutoff point corresponding to five times the reference value for normal adults, has been used in clinical practice [14]. Prematurity is one of the cause of early neonatal hypocalcemia (within 48–72 h of birth). Possible mechanisms include poor intake, decreased responsiveness to vitamin D, increased calcitonin, and hypoalbuminemia leading to decreased total but normal ionized calcium [15].

In humans, rapid development of important functional cell structures in the lungs, pancreas, and kidneys takes place until the last few weeks of gestation and preterm birth may affect final development [16, 17]. It has been suggested that premature birth impairs final development of nephrons (after birth) [18, 19]. Nephron number was highly correlated to gestational age. Therefore, a reduced nephron number after preterm birth persists throughout life and may affect long-term renal function and blood pressure [20, 21].

## 2. Material and Method

75 babies admitted to Department of Pediatrics, and its neonatal unit was enrolled for the present study. The enrolled neonates were further divided into study group (50 neonates) and control group (25 neonates). 12 hr fasting blood samples were collected in EDTA vials, and serum calcium, phosphorus, alkaline phosphatase, creatinine, sodium, and potassium were measured in three different groups of preterm babies at 28–30 weeks, 30–32, weeks and 34–36 weeks of GA.

Serum calcium was estimated by OCPC method.
Serum phosphorus was estimated by Modified Metol method.
Serum alkaline phosphatase was estimated by Kinetic p-NPP method.
Serum creatinine was estimated by Jaffes method.
Serum Na^+^ and K^+^ was measured by Electrolyte analyzer.

Student “*t*-test” will be applied to calculate the significance in differences of these parameters between the groups. Regression analysis will be done to study the interrelation between the said parameters. All calculation will be done by SPSS 9 software.

## 3. Result and Discussion

The present study was conducted in neonatal intensive care unit of Department of Pediatrics, Kamla Nehru Hospital, in collaboration with Department of Biochemistry, Gandhi Medical College, Bhopal, from January 2008 to January 2009.

Of the 75 preterm babies enrolled, 45% were males and 30% females, and there is 37 mothers of preterm babies were of a poor dietary intake, whereas 20 mothers were of an average dietary intake and 18 mothers were of a normal dietary intake.

The main findings of the study were as follows.

**Figure 1:**
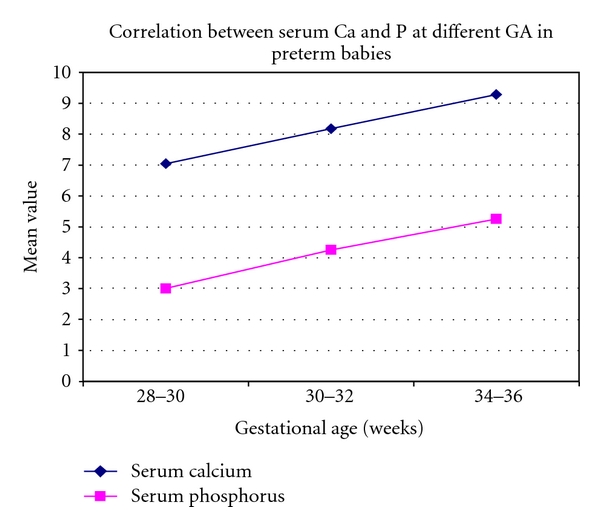
This figure indicates that there is a positive correlation between serum calcium & phosphorus levels. The values are increases parallel with the advancement of GA.

**Figure 2:**
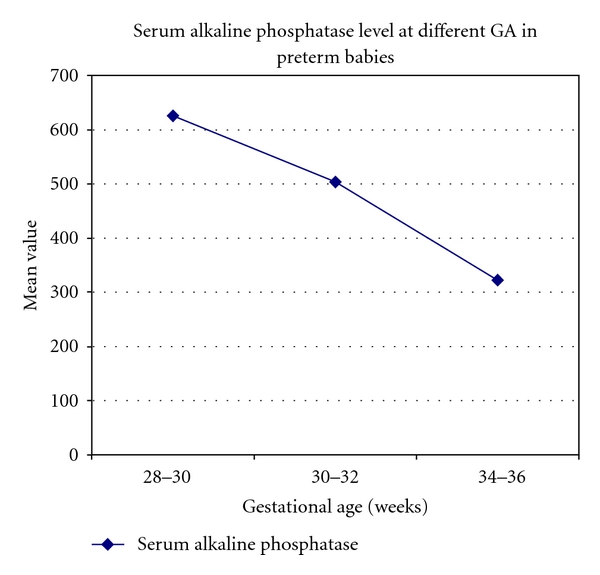
This figure indicates that the activity of alkaline phosphatase is decreases with the advancement of GA.

1. Table 1 shows that Serum calcium and phosphorus levels were significantly decreased at 28–30 weeks as compared to 34–36 weeks (Controls), but it was insignificantly decreased at 28–30 weeks as compared to 30–32 weeks and at 30–32 weeks as compared to 34–36 weeks (Controls) (as Table 2 shows) of gestational age in preterm babies.
2. Tables 1 and 2 show that Serum alkaline phosphatase level was significantly increased at 28–30 weeks and 30–32 weeks as compared to 34–36 weeks of gestational age in preterm babies, but it was insignificantly increased at 28–30 weeks as compared to 30–32 weeks (Table 3 shows) in preterm babies.
3. Serum calcium and phosphorus level in preterm babies whose mothers were fed calcium and phosphorus rich diet was found to be significantly higher than that found in preterm babies whose mothers were not fed calcium and phosphorus rich diet.
4. Figure 1 shows that the correlation between serum calcium and phosphorus were found to be positive at all gestational ages. The values were increases parallely with the advancement of GA in preterm babies.
5. Figure 2 shows that the activity of serum alkaline phosphatase is inversely correlated with serum calcium and phosphorus at all gestational ages. As the gestational ages were increased, the activity of serum alkaline phosphatase was significantly decreased in preterm babies.
6. Tables 1 and 2 show that serum creatinine was significantly increased at 28–30 weeks and 30–32 weeks as compared to 34–36 weeks of gestational age in preterm babies, but it was insignificantly increased at 28–30 weeks as compared to 30–32 weeks in preterm babies.
7. Tables 1, 2, and 3 show that the serum sodium and potassium levels were insignificantly increased at all gestational ages in preterms.
8. There is a negative correlation between serum creatinine, sodium, potassium, and the gestational age.

Decreased serum calcium and phosphorus levels in preterm babies signifies inadequate calcium and phosphate intake, reduced opportunity for transplacental mineral delivery and excessive mineral loss after birth in preterm babies, decreased bone mineralization and increased bone resorption, increased calcitonin, and increased urinary calcium and phosphorus excretion in preterm babies. Increased alkaline phosphatase level signifies increased bone cellular or osteoblastic activity in preterm babies [22]. Increased serum creatinine and electrolytes signifies lower GFR in preterms. Glomerular function shows a progression directly correlated to GA and postnatal age in preterm infants [23].

## Figures and Tables

**Table 1:** Comparison of serum calcium, phosphorus, alkaline phosphatase, creatinine and electrolyte levels at 28–30 weeks and 34–36 weeks (Controls) of gestational age in preterm babies.

S. No	Parameter	Cases (*n* = 25)	Controls (*n* = 25)	*P*-Value
		28–30 weeks	34–36 weeks	
(1)	Serum Calcium (mg/dL)	7.044 ± 1.753	9.284 ± 1.276	<0.001
(2)	Serum Phosphorus (mg/dL)	3.012 ± 0.799	5.256 ± 1.308	<0.001
(3)	Serum Alkaline Phosphatase (IU/L)	625.56 ± 176.28	322.08 ± 80.07	<0.001
(4)	Serum Creatinine (mmol/L)	67.64 ± 7.4	46.31 ± 7.7	<0.001
(5)	Serum Sodium (mmol/L)	136.6 ± 4.1	132.8 ± 3.8	NS
(6)	Serum Potassium (mmol/L)	6.98 ± 0.72	5.36 ± 0.60	<0.01

It is evident from this table that serum calcium and phosphorus level is decreased significantly whereas alkaline phosphatase and creatinine levels are significantly and electrolytes are non-significantly increased at 28–30 weeks as compared to 34–36 weeks of gestational age in preterm babies.

**Table 2:** Comparison of serum calcium, phosphorus, alkaline phosphatase, creatinine and electrolyte levels at 30–32 weeks and 34–36 weeks (Controls) of gestational age in preterm babies.

S. No	Parameter	Cases (*n* = 25)	Control (*n* = 25)	*P*-Value
		30–32 weeks	34–36 weeks	
(1)	Serum Calcium (mg/dL)	8.176 ± 1.771	9.284 ± 1.276	NS
(2)	Serum Phosphorus (mg/dL)	4.256 ± 1.126	5.256 ± 1.308	NS
(3)	Serum Alkaline Posphatase (IU/L)	503.48 ± 164.37	322.08 ± 80.07	<0.001
(4)	Serum Creatinine (mmol/L)	58.52 ± 3.8	46.31 ± 7.7	<0.001
(5)	Serum Sodium (mmol/L)	134.04 ± 2.71	132.8 ± 3.8	NS
(6)	Serum Potassium (mmol/L)	6.16 ± 0.48	5.36 ± 0.60	NS

This table shows that serum calcium & phosphorus levels is decreased (statistically insignificant) whereas alkaline phosphatase and creatinine levels are significantly and electrolytes are non-significantly increased at 30–32 weeks as compared to 34–36 weeks of gestational age in preterm babies.

**Table 3:** Comparison of serum calcium, phosphorus, alkaline phosphatase, creatinine and electrolyte levels at 28–30 weeks and 30–32 weeks of gestational age in preterm babies.

S. No.	Parameters	Cases (*n* = 25)	Cases (*n* = 25)	*P*-Value
		28–30 weeks	30–32 weeks	
(1)	Serum Calcium (mg/dL)	7.044 ± 1.753	8.176 ± 1.771	NS
(2)	Serum Phosphorus (mg/dL)	3.012 ± 0.799	4.256 ± 1.126	NS
(3)	Serum Alkaline Phosphatase (IU/L)	625.56 ± 176.28	503.48 ± 164.37	NS
(4)	Serum Creatinine (mmol/L)	67.64 ± 7.4	58.52 ± 3.8	NS
(5)	Serum Sodium (mmol/L)	136.6 ± 4.1	134.04 ± 2.71	NS
(6)	Serum Potassium (mmol/L)	6.98 ± 0.72	6.16 ± 0.48	<0.01

It is evident from this table that serum calcium & phosphorus levels are decreased (statistically insignificant) whereas alkaline phosphatase, creatinine and electrolytes level are insignificantly increased at 28–30 weeks as compared to 30–32 weeks of gestational age in preterm babies.

## Abbreviations

MBDP: Metabolic bone disease of prematurity
Ca: Calcium
Pi: Inorganic phosphate
ALP: Alkaline phosphatase
BMD: Bone mineral density
VLBW: Very low birth weight
CT: Calcitonin
ELBW: Early low birth weight
PT: Preterm
GA: Gestational age
GFR: Glomerular filtration rate.
